# The Radiological Assessment of Carotid Space Lesions: A Case Series

**DOI:** 10.7759/cureus.62202

**Published:** 2024-06-11

**Authors:** Stany Jerosha, Sakthi Ganesh Subramonian, Karthik Krishna Ramakrishnan

**Affiliations:** 1 Radiodiagnosis, Saveetha Medical College and Hospital, Saveetha Institute of Medical and Technical Sciences, Saveetha University, Chennai, IND

**Keywords:** multidisciplinary approach, diagnostic challenges, schwannoma, inflammatory pseudotumor, radiology, carotid body tumor, carotid space lesion

## Abstract

Carotid space lesions present diagnostic challenges due to their diverse etiology and varied clinical manifestations. This article critically reviews the anatomy of the carotid space and highlights the spectrum of pathologies within this complex region, illustrated by three case studies. The cases were examined with ultrasonography (USG), computed tomography (CT), and magnetic resonance imaging (MRI). Schwannomas appeared heterogeneously hypodense on plain CT and partially hyperdense on contrast-enhanced CT (CECT), with displacement of adjacent vessels. Vagal-origin schwannomas caused the anteromedial displacement of the internal carotid artery. Paragangliomas were typically homogeneously hyperdense on CECT, with lateral displacement of the internal carotid artery when of carotid body origin. The management of carotid space lesions depends on the resectability of the tumors; unresectable tumors are managed with chemotherapy. This overview enhances clinical understanding and diagnostic accuracy, facilitating improved patient outcomes in managing carotid space lesions.

## Introduction

The carotid space, a distinct anatomical region within the neck, encompasses a variety of pathologies due to its complex structure confined within a small area [1]. This critical review delineates the intricate anatomy of the carotid space, extending from the skull base to the thorax [1,2], and highlights the diverse mass lesions and vascular anomalies that may arise therein. By elaborating on the carotid space's anatomy, including its borders and contents across various levels, this article provides an in-depth examination of mass lesions such as paragangliomas, nerve sheath tumors, and others, alongside vascular pathologies like carotid dissection and pseudoaneurysm [2]. The discussion is enriched by insights into anatomic considerations crucial for differential diagnoses [3], imaging features, and characteristics of lesions across multiple imaging modalities including computed tomography (CT), magnetic resonance imaging (MRI), ultrasonography (USG), and conventional angiography. This comprehensive overview not only enhances the understanding of the carotid space's unique anatomy but also empowers healthcare professionals with the knowledge to accurately diagnose the wide spectrum of pathologies it may harbor, facilitating improved patient outcomes.

## Case presentation

Case 1

**Figure 1:**
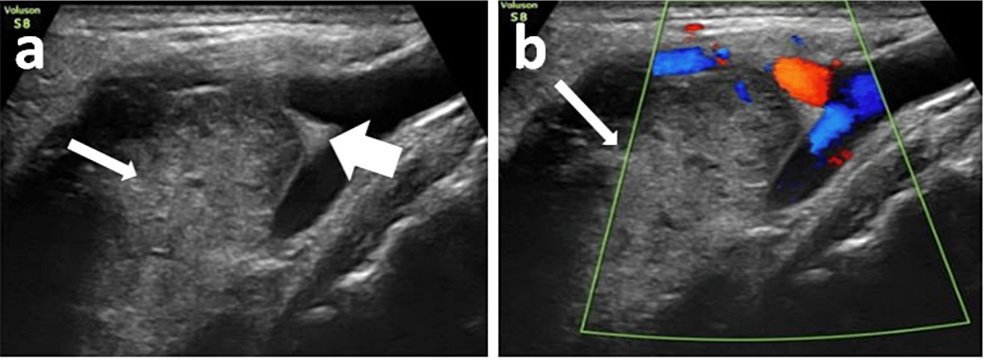
Ultrasound images of the neck demonstrating a lesion at the bifurcation of the common carotid artery (CCA). (a) USG neck (longitudinal orientation) showing a well-defined relatively heterogeneous iso- to hyperechoic to muscle lesion (long white arrow) in the region of bifurcation of CCA, causing splaying of ECA and ICA (short white arrow). (b) The lesion (long white arrow) does not show internal vascularity (Probe marker: Voluson S8; GE Healthcare, Chicago, USA). ECA: external carotid artery; ICA: internal carotid artery

A 25-year-old male presented with intermittent palpitations and vomiting for two months, along with a headache that started a month ago and pain below the right submandibular region persisting for one month. The patient has recently been diagnosed with hypertension and commenced treatment with Nicardia, taken once daily. There was no significant family history. On physical examination, a firm, smooth, 3 x 2 cm swelling was noted in the right inguinal region, with no warmth, erythema, or tenderness. Additionally, a 2 x 3 cm swelling was observed in the right submandibular region, characterized by its firm consistency and smooth surface, though tenderness was present. Multiple enlarged lymph nodes were noted along the right sternocleidomastoid muscle (SCM). The patient’s blood pressure was elevated to 150/100 mmHg, with all other vitals within normal ranges. Laboratory investigations highlighted elevated blood catecholamine levels, while other tests did not show any abnormalities. USG showed a well-defined heterogeneous lesion of size 5 x 5 x 5.3 cm in the region of bifurcation of the common carotid artery (CCA), causing splaying of external carotid artery (ECA) and internal carotid artery (ICA) and had compression effect over ICA and ECA (Figure 1).

**Figure 2:**
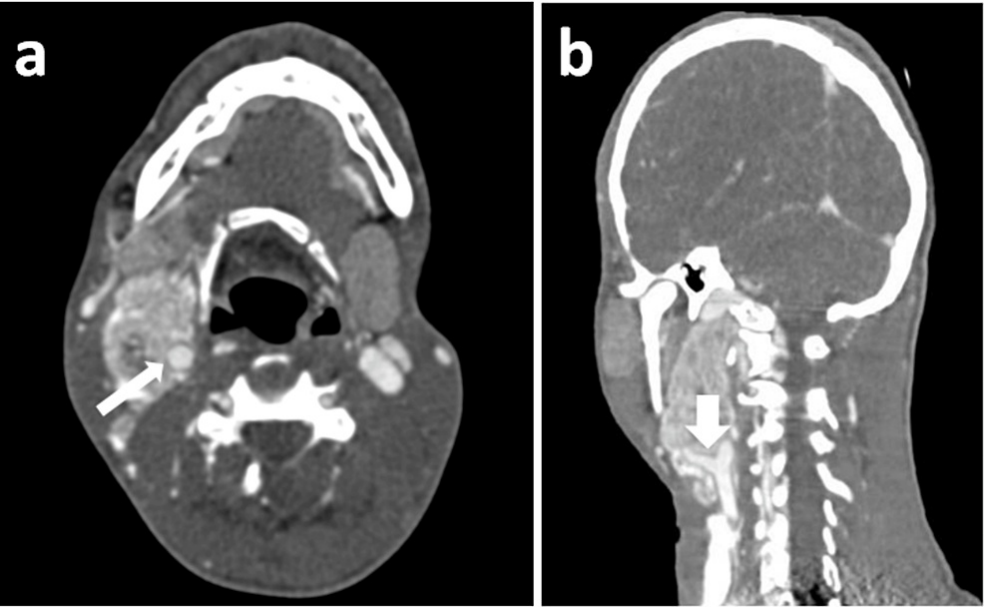
Computed tomography images of the neck demonstrating a lesion in the right carotid space. (a) CECT neck axial section showing a heterogeneous, predominantly hyper-enhancing soft tissue dense lesion epicentered in the right carotid space causing encasement of the internal carotid artery - Shamblin group III (long white arrow). (b) CECT neck coronal section showing heterogeneously enhancing soft tissue dense lesion causing splaying of ECA and ICA (short white arrow). CECT: contrast-enhanced computed tomography; ECA: external carotid artery; ICA: internal carotid artery

Contrast-enhanced CT (CECT) revealed a heterogeneously enhancing soft tissue dense lesion measuring 2.5 x 5.2 x 6.2 cm epicentered in the right carotid space (at the level of hyoid bone) causing encasement of internal and external carotid arteries and its branches (Figure 2).

**Figure 3:**
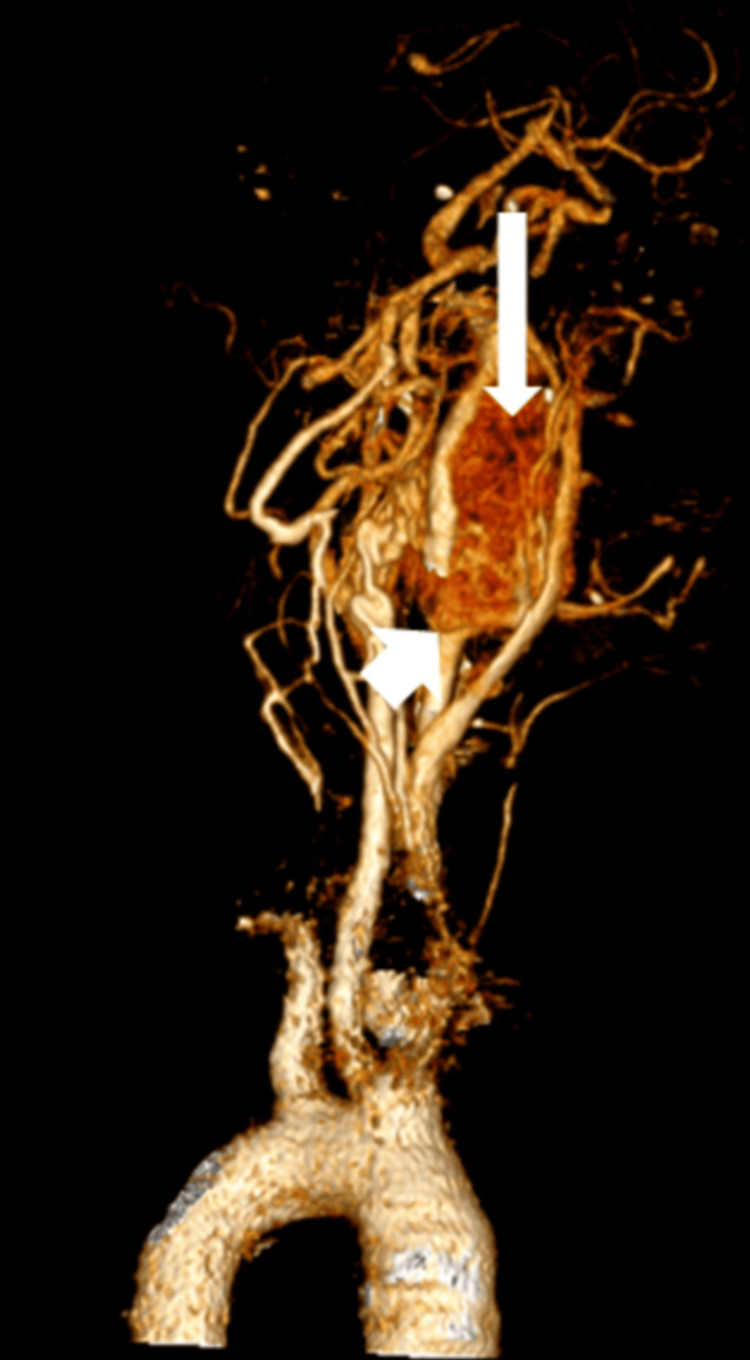
Volume-rendered 3D reconstruction of CECT neck Volume-rendered 3D reconstruction of CECT neck showing mass lesion (long white arrow) causing splaying of ECA and ICA (short white arrow). CECT: contrast-enhanced computed tomography; ECA: external carotid artery; ICA: internal carotid artery

The lesion was categorized under the Shamblin group system as group III: >270 degrees of encasement. Volume-rendered 3D reconstruction was done to demonstrate the splaying of ECA and ICA (Figure 3).

The lesion was surgically excised, and a histopathological examination of the specimen confirmed the diagnosis of paraganglioma.

Case 2

A 40-year-old female presented with progressive, painless swelling on the left side of her neck, noted over the past six months. She reported occasional episodes of palpitations and unexplained bouts of sweating, especially at night. There was no significant family history. On examination, a palpable, firm, and non-tender mass was noted at the left carotid bifurcation, without overlying skin changes. Vital signs were within normal limits except for a slightly elevated blood pressure of 145/90 mmHg. The remainder of the physical examination was unremarkable. Further biochemical analysis revealed elevated plasma catecholamine levels.

**Figure 4:**
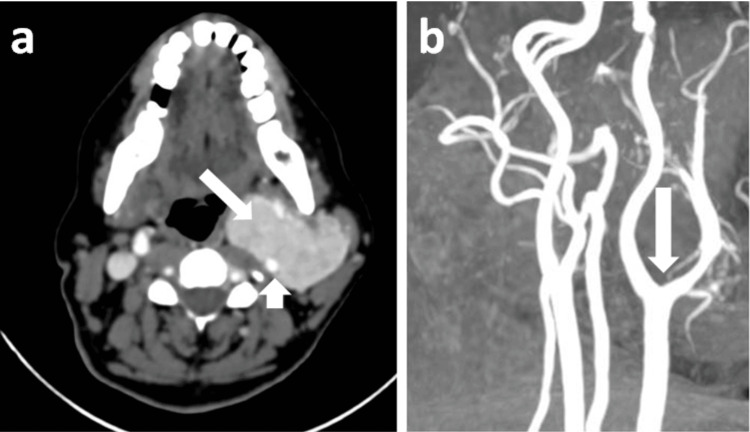
Computed tomography images of the neck demonstrating a lesion in the left carotid space. (a) CECT neck axial section showing heterogeneous, predominantly hyper-enhancing soft tissue dense lesion (long white arrow) epicentered in the left carotid space at the level of bifurcation of left CCA causing encasement of the cervical segment of left ICA - Shamblin group III (short white arrow). (b) CT angiography of the neck showing splaying of left ECA and ICA (long white arrow). CECT: contrast-enhanced computed tomography; ECA: external carotid artery; ICA: internal carotid artery; CCA: common carotid artery

CT revealed a heterogeneously enhancing soft tissue dense lesion measuring 2.5 x 5.2 x 6.2 cm epicentered in the left carotid space (at the level of bifurcation of left CCA) causing encasement of left CCA and cervical segment of right ICA and splaying of ECA and ICA (Figure 4).

**Figure 5:**
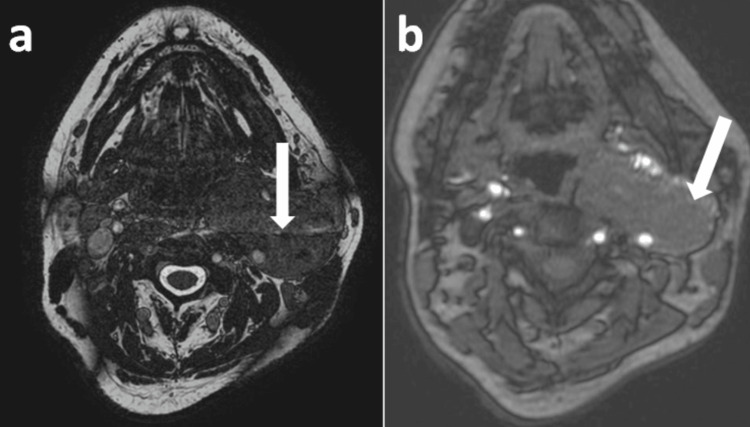
MRI images of the neck demonstrating a lesion in the left carotid space. (a) MRI T2-weighted image axial section showing hyperintense lesion (long white arrow) epicentered in the left carotid space. (b) Magnetic resonance angiography (MRA) neck image axial section showing muscle isointense lesion (long white arrow) epicentered in the left carotid space causing splaying of ECA and ICA. ECA: external carotid artery; ICA: internal carotid artery

The lesion was categorized under the Shamblin group system as group III: >270 degrees of encasement. MRI revealed a soft tissue intense lesion measuring 2.5 x 5.2 x 6.2 cm epicentered in the left carotid space (at the level of hyoid bone) causing encasement of the cervical segment of the left ICA and splaying the left external and internal carotid arteries (Figure 5).

The lesion was surgically excised, and a histopathological examination of the specimen confirmed the diagnosis of paraganglioma.

Case 3

A 47-year-old female patient presented complaints of a painless neck swelling that had been gradually increasing in size over the past six months. There was no relevant family history. On physical examination, a firm, smooth, non-tender swelling measuring 5 x 3 cm was observed in the right submandibular region, without any signs of warmth or erythema. Additionally, right-sided lymphadenopathy was noted, with multiple lymph nodes palpable along the SCM. Vital signs showed an elevated blood pressure of 150/100 mmHg, with all other vitals within normal ranges. Laboratory investigations did not reveal any abnormalities.

**Figure 6:**
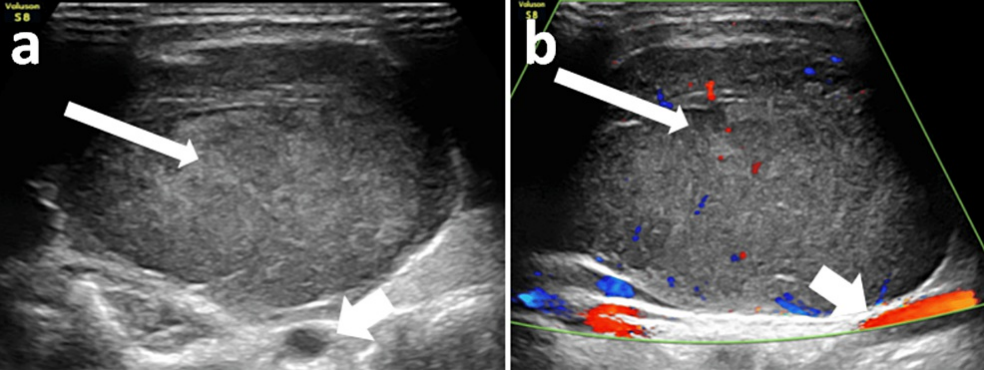
Ultrasound images of the neck demonstrating a lesion anterior to the right internal carotid artery. (a) USG neck (longitudinal orientation) showing relatively heterogenous lesion (long white arrow) anterior to the right internal carotid artery (short white arrow). (b) USG neck colour Doppler shows mild internal vascularity within the lesion (long white arrow) and colour uptake within the internal carotid artery (short white arrow) (Probe marker: Voluson S8).

USG revealed a well-defined homogeneous lesion of size 5 x 3.6 x 7.3 cm just anterior to the right ICA. No splaying of carotid vessels was observed, with displacement of the carotid vessels medially (Figure 6).

**Figure 7:**
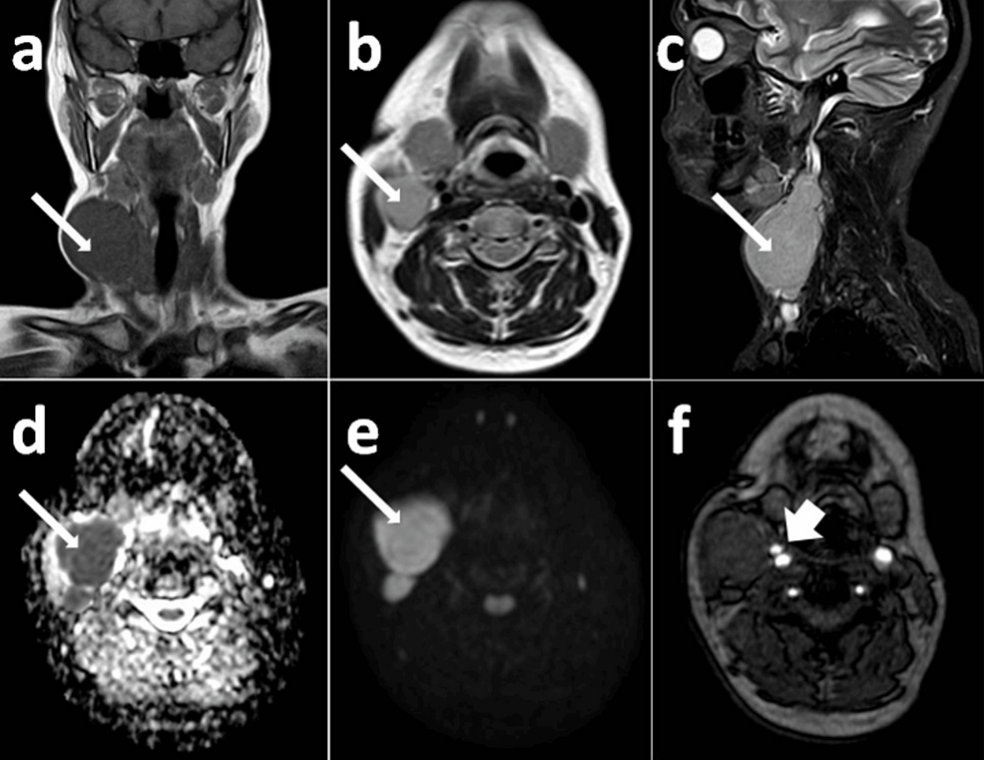
MRI images of the neck demonstrating a lesion in the right carotid space. (a) MRI T1-weighted image coronal section showing a well-defined T1 hypointense lesion (long white arrow) epicentered in the right carotid space. (b) MRI T2-weighted image axial section showing a well-defined T2 hyperintense to muscle lesion (long white arrow) epicentered in the right carotid space. (c) MRI STIR image sagittal section showing a well-defined hyperintense to muscle lesion (long white arrow) epicentered in the right carotid space. (d) ADC map of the neck shows low ADC levels involving the lesion (long white arrow) epicentered in the right carotid space. (e) The lesion shows corresponding diffusion restriction (long white arrow). (f) MRA axial section showing medial displacement of right internal and external carotid vessels (short white arrow). STIR: short tau inversion recovery; ADC: apparent diffusion coefficient; MRA: magnetic resonance angiography

MRI revealed T1 hypointense/T2 hyperintense and short tau inversion recovery (STIR) isointense lesion measuring 4.6 x 4.3 x 6.3 cm epicentered in the right carotid space compressing the right internal jugular vein (IJV) and pushing the carotid vessels medially. The lesion shows diffusion restriction with a corresponding low apparent diffusion coefficient (ADC) signal (Figure 7).

Fine needle aspiration and cytology (FNAC) confirmed the diagnosis of vagal schwannoma, following which the patient was suggested surgical management; however, the patient denied surgery and hence was started on chemotherapy.

## Discussion

Anatomy of the carotid space

The carotid space encompasses the area from the inferior margin of the jugular foramen-carotid canal to the aortic arch, bordered by the three layers of the deep cervical fascia, which contains the CCA, IJV, vagus nerve, and sympathetic chain. This space comprises both suprahyoid and infrahyoid regions.

The suprahyoid portion of the carotid space contains the ICA, the IJV, cranial nerves 9 through 12, the ansa cervicalis, the sympathetic plexus, and deep cervical lymph nodes [1]. The infrahyoid portion contains the ansa cervicalis, cranial nerve 10, IJV, and the CCA [2].

Classification of carotid space lesions

Carotid Body Tumors (CBTs)

Arising from paraganglionic tissue at the carotid bifurcation [3], CBTs are typically benign but may exhibit malignant behavior. Clinical presentation varies from asymptomatic to compressive symptoms. On USG, CBTs appear as well-defined, hypoechoic masses with rich vascularity on Doppler. CT demonstrates a hypervascular mass with calcifications, while MRI shows a well-defined lesion with high T2 signal intensity.

Schwannomas

Derived from Schwann cells of the cervical sympathetic chain, schwannomas are encapsulated benign tumors. They often present with gradual enlargement and compressive symptoms [4]. USG reveals a hypoechoic, well-circumscribed mass with posterior acoustic enhancement. CT demonstrates a well-defined, homogeneously enhancing lesion, while MRI shows low-to-intermediate signal intensity on T1-weighted images and high signal intensity on T2-weighted images.

Paragangliomas

They arise from paraganglionic tissue along the sympathetic chain or within the carotid body [5]. These tumors may exhibit hormonal secretion and carry a risk of metastasis.

Inflammatory Pseudotumor (IPT)

Characterized by chronic inflammatory infiltrates, IPTs mimic neoplasms clinically and radiologically [6]. They pose diagnostic challenges due to their diverse presentations and variable imaging features. USG demonstrates a hypoechoic, poorly defined mass with heterogeneous echotexture. CT reveals a poorly defined, heterogeneously enhancing lesion, while MRI shows variable signal intensity depending on the degree of fibrosis and inflammation.

Lymphoepithelial Cysts

They result from branchial cleft remnants or lymphatic tissue [7]. These cystic lesions may mimic neoplasms on imaging and present with compressive symptoms.

Carotid Artery Aneurysms

Focal dilatations result from the weakening of the arterial wall. They may present with pulsatile masses and compressive symptoms [8]. USG demonstrates a cystic or complex hypoechoic mass adjacent to the carotid artery. CT reveals a well-defined, contrast-enhancing lesion with calcifications, while MRI shows flow voids within the aneurysm.

Carotid Artery Dissections

Carotid artery dissections occur due to intimal tears with subsequent hematoma formation, resulting in varying degrees of stenosis or occlusion [9]. Clinical presentation ranges from asymptomatic to acute neurological deficits. Radiological imaging, including ultrasound, CT, MRI, and positron emission tomography-computed tomography (PET-CT), plays a pivotal role in characterizing carotid space lesions [10]. FNAC provides valuable diagnostic information [11], although histopathological examination remains the gold standard for definitive diagnosis.

Diagnostic Modalities

Radiological imaging, including ultrasound, CT, MRI, and PET-CT, plays a pivotal role in characterizing carotid space lesions. FNAC provides valuable diagnostic information, although histopathological examination remains the gold standard for definitive diagnosis.

PET-CT

18F-FDOPA (Fluorodopa): Provides excellent lesion contrast and is especially useful for head and neck paragangliomas.

68Ga-DOTATATE (Gallium-68 DOTATATE): High sensitivity and specificity, particularly for well-differentiated tumors.

18F-FDA (Fluorodopamine): Excellent for imaging of pheochromocytomas and paragangliomas.

Shamblin's Classification

**Figure 8:**
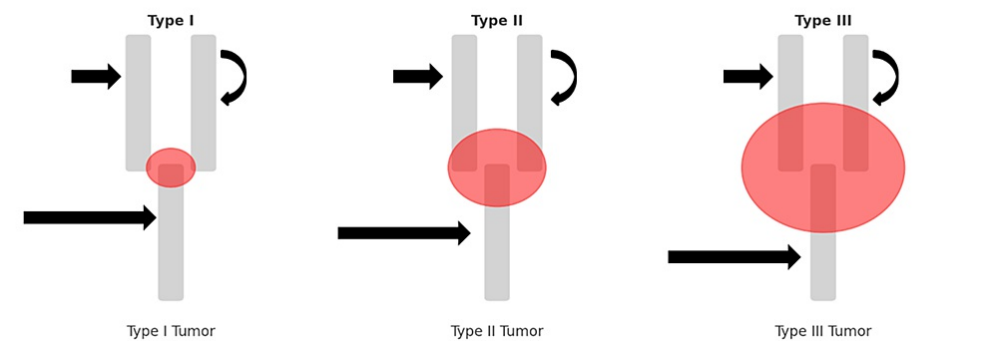
Illustration of Shamblin's classification of carotid body tumors. Type I tumors are small and minimally attached to the carotid artery; Type II tumors are larger and partially encircle the artery; and Type III tumors are extensive, fully encasing the artery and often involving nearby nerves and vessels (long black arrow - common carotid artery; short black arrow - internal carotid artery; curved black arrow - external carotid artery). The image was created by the authors.

Shamblin's classification is a system for categorizing CBTs based on their size and involvement with surrounding tissues. Created by Dr. John Shamblin [12] in the 1970s, it assists in surgical planning and prognosis. There are three types: Type I tumors are small and minimally attached to the carotid artery; Type II tumors are larger and partially encircle the artery; and Type III tumors are extensive, fully encasing the artery and often involving nearby nerves and vessels. This classification helps gauge the complexity of surgical removal and the associated risks (Figure 8).

The displacement pattern of the carotid artery and IJV can help in narrowing down the differential diagnosis (Table 1).

**Table 1:** Differential diagnoses for carotid space lesions based on the displacement of the carotid artery and internal jugular vein. This table provides an overview of the differential diagnoses for carotid space lesions, focusing on the characteristic displacement of nearby vascular structures. This information can assist in narrowing down the differential diagnosis based on imaging findings.

Lesion Type	Displacement of Vessels	Common Features
Paraganglioma (Carotid Body Tumor)	Splaying of the carotid bifurcation (Lyre sign)	Highly vascular, enhancing imaging
Schwannoma	Anteromedial displacement of carotid artery	Homogeneous, slow-growing, well-defined, may cause nerve symptoms
Neurofibroma	Anteromedial displacement of carotid artery	Associated with neurofibromatosis, variable enhancement
Metastatic Lymphadenopathy	Variable displacement depending on size and location	Often multiple, irregular enhancement
Abscess	Anteromedial displacement of carotid artery	Rim-enhancing lesion, signs of infection
Carotid Artery Aneurysm	Displacement variable may be minimal	Fusiform or saccular dilatation of the artery
Glomus Vagale	Posterolateral displacement of carotid artery	Vascular mass at the skull base, similar to paraganglioma
Lymphoma	Variable displacement depending on size and location	Homogeneous, often encasing vessels
Meningioma	May encase and displace carotid artery	Dural attachment, enhancing with possible calcifications
Salivary Gland Tumors (e.g., Pleomorphic Adenoma)	Anteromedial displacement of vessels	Well-circumscribed, arising from the deep lobe of parotid
Branchial Cleft Cyst	Lateral displacement of the carotid artery	Cystic mass with possible infection or fistula

Management Strategies

Management of carotid space lesions is tailored to the specific pathology, patient factors, and lesion characteristics [13]. Surgical excision remains the cornerstone for resectable lesions [14], aiming for complete removal while preserving neurovascular structures. Adjuvant therapies, including radiotherapy and embolization, may be employed in selected cases, particularly for unresectable or malignant lesions [15,16].

Review of literature

Various published literature on carotid space lesions have been reviewed and compiled in Table 2.

**Table 2:** Summary of literature review

References	Topics	Key Points	Significances
Gervasio et al. (2011) [1]	Sonographic anatomy of the neck: suprahyoid region	Detailed sonographic anatomy; ultrasound utility in identifying anatomical landmarks and pathologies	Enhances understanding of suprahyoid region anatomy and supports diagnostic accuracy with ultrasound
Kuwada et al. (2012) [2]	Imaging of the carotid space	Comprehensive guide on carotid space anatomy; roles of computed tomography, magnetic resonance imaging, and ultrasound; imaging characteristics of common pathologies	Provides a framework for evaluating carotid space lesions using multiple imaging modalities
Lee et al. (2006) [3]	Imaging features of extra-adrenal paragangliomas	Imaging characteristics of paragangliomas; use of computed tomography, magnetic resonance imaging, and other imaging techniques	Helps in distinguishing paragangliomas from other lesions based on imaging features
Roman (2004) [4]	Pheochromocytoma and functional paraganglioma	Clinical presentation, diagnosis, and management of pheochromocytomas and functional paragangliomas	Essential for understanding the clinical management of these functional tumors
Sridhara et al. (2013) [5]	Genetic testing in head and neck paraganglioma	Importance of genetic testing; who should be tested, what tests to perform, and why	Highlights the role of genetics in diagnosis and management, aiding personalized treatment approaches
Woolen and Gemmete (2016) [6]	Paragangliomas of the head and neck	Imaging techniques and characteristics; clinical features of paragangliomas	Provides detailed imaging findings and clinical correlations for head and neck paragangliomas
Gervasio et al. (2010) [7]	Ultrasound anatomy of the neck: infrahyoid region	Detailed sonographic anatomy; ultrasound utility in identifying anatomical landmarks and pathologies	Supports diagnostic accuracy and understanding of infrahyoid region anatomy
Saurborn et al. (2003) [8]	Paraganglioma of the organs of Zuckerkandl	Case study on paragangliomas; imaging and diagnostic approach	Illustrates the diagnostic process and imaging features of rare paragangliomas
Dunnick and Korobkin (2002) [9]	Imaging of adrenal incidentalomas	Current imaging techniques; incidental findings and their management	Guides clinicians on handling incidental adrenal findings and their implications
Vanderveen et al. (2009) [10]	Biopsy of pheochromocytomas and paragangliomas	Risks associated with biopsy; recommendations for safe practices	Advises on the potential dangers of biopsying these tumors and safer diagnostic alternatives
Davidovic et al. (2005) [11]	Diagnosis and treatment of carotid body paraganglioma	Clinical experience; diagnostic and treatment strategies over 21 years	Provides long-term clinical insights into the management of carotid body paragangliomas
Gray et al. (1967) [13]	Gray’s anatomy: descriptive and applied	Fundamental anatomical descriptions; comprehensive reference on human anatomy	Serves as a foundational anatomical reference for medical professionals
Rao et al. (1999) [14]	Imaging of peripheral nerve sheath tumors	Characteristic imaging signs on computed tomography, magnetic resonance imaging, and sonography	Aids in the identification and differentiation of peripheral nerve sheath tumors based on imaging
Isobe et al. (1994) [15]	Case report on “ancient” schwannoma	Imaging and clinical features of degenerated schwannomas	Provides insights into the imaging and clinical presentation of rare, aged schwannomas
Lin and Martel (2001) [16]	Cross-sectional imaging of peripheral nerve sheath tumors	Computed tomography, magnetic resonance imaging, and sonography features; characteristic imaging signs	Enhances diagnostic capabilities for peripheral nerve sheath tumors through detailed imaging descriptions

## Conclusions

Carotid space lesions encompass a spectrum of pathologies, necessitating a nuanced approach to diagnosis and management. This multicase report underscores the importance of recognizing diverse etiologies beyond CBTs and delineating individual lesion characteristics through a comprehensive evaluation. Through interdisciplinary collaboration and understanding of lesion pathophysiology, optimal patient outcomes can be achieved.

## References

[1] Gervasio A, D'Orta G, Mujahed I, Biasio A (2011). Sonographic anatomy of the neck: the suprahyoid region. J Ultrasound.

[2] Kuwada C, Mannion K, Aulino JM, Kanekar SG (2012). Imaging of the carotid space. Otolaryngol Clin North Am.

[3] Lee KY, Oh YW, Noh HJ (2006). Extraadrenal paragangliomas of the body: imaging features. AJR Am J Roentgenol.

[4] Roman S (2004). Pheochromocytoma and functional paraganglioma. Curr Opin Oncol.

[5] Sridhara SK, Yener M, Hanna EY, Rich T, Jimenez C, Kupferman ME (2013). Genetic testing in head and neck paraganglioma: who, what, and why?. J Neurol Surg B Skull Base.

[6] Woolen S, Gemmete JJ (2016). Paragangliomas of the head and neck. Neuroimaging Clin N Am.

[7] Gervasio A, Mujahed I, Biasio A, Alessi S (2010). Ultrasound anatomy of the neck: the infrahyoid region. J Ultrasound.

[8] Saurborn DP, Kruskal JB, Stillman IE, Parangi S (2003). Best cases from the AFIP: paraganglioma of the organs of Zuckerkandl. Radiographics.

[9] Dunnick NR, Korobkin M (2002). Imaging of adrenal incidentalomas: current status. AJR Am J Roentgenol.

[10] Vanderveen KA, Thompson SM, Callstrom MR (2009). Biopsy of pheochromocytomas and paragangliomas: potential for disaster. Surgery.

[11] Davidovic LB, Djukic VB, Vasic DM, Sindjelic RP, Duvnjak SN (2005). Diagnosis and treatment of carotid body paraganglioma: 21 years of experience at a clinical center of Serbia. World J Surg Oncol.

[12] Arya S, Rao V, Juvekar S, Dcruz AK (2008). Carotid body tumors: objective criteria to predict the Shamblin group on MR imaging. AJNR Am J Neuroradiol.

[13] Gray H, Davies DV, Coupland RE (1967). Gray’s Anatomy: Descriptive and Applied.

[14] Rao AB, Koeller KK, Adair CF (1999). From the archives of the AFIP. Paragangliomas of the head and neck: radiologic-pathologic correlation. Armed Forces Institute of Pathology. Radiographics.

[15] Schultz E, Sapan MR, McHeffey-Atkinson B, Naidich JB, Arlen M (1994). Case report 872. "Ancient" schwannoma (degenerated neurilemoma). Skeletal Radiol.

[16] Lin J, Martel W (2001). Cross-sectional imaging of peripheral nerve sheath tumors: characteristic signs on CT, MR imaging, and sonography. AJR Am J Roentgenol.

